# Transcriptome analysis reveals the potential roles of long non-coding RNAs in feed efficiency of chicken

**DOI:** 10.1038/s41598-022-06528-6

**Published:** 2022-02-15

**Authors:** Parastoo Karimi, Mohammad Reza Bakhtiarizadeh, Abdolreza Salehi, Hamid Reza Izadnia

**Affiliations:** 1 Department of Animal and Poultry Science, College of Aburaihan, University of Tehran, Tehran, Iran; 2 Animal Science Improvement Research Department, Agricultural and Natural Resources Research and Education Center, Safiabad AREEO, Dezful, Iran

**Keywords:** Gene expression profiling, Gene regulatory networks

## Abstract

Feed efficiency is an important economic trait and reduces the production costs per unit of animal product. Up to now, few studies have conducted transcriptome profiling of liver tissue in feed efficiency-divergent chickens (Ross vs native breeds). Also, molecular mechanisms contributing to differences in feed efficiency are not fully understood, especially in terms of long non-coding RNAs (lncRNAs). Hence, transcriptome profiles of liver tissue in commercial and native chicken breeds were analyzed. RNA-Seq data along with bioinformatics approaches were applied and a series of lncRNAs and target genes were identified. Furthermore, protein–protein interaction network construction, co-expression analysis, co-localization analysis of QTLs and functional enrichment analysis were used to functionally annotate the identified lncRNAs. In total, 2,290 lncRNAs were found (including 1,110 annotated, 593 known and 587 novel), of which 53 (including 39 known and 14 novel), were identified as differentially expressed genes between two breeds. The expression profile of lncRNAs was validated by RT-qPCR. The identified novel lncRNAs showed a number of characteristics similar to those of known lncRNAs. Target prediction analysis showed that these lncRNAs have the potential to act in cis or trans mode. Functional enrichment analysis of the predicted target genes revealed that they might affect the differences in feed efficiency of chicken by modulating genes associated with lipid metabolism, carbohydrate metabolism, growth, energy homeostasis and glucose metabolism. Some gene members of significant modules in the constructed co-expression networks were reported as important genes related to feed efficiency. Co-localization analysis of QTLs related to feed efficiency and the identified lncRNAs suggested several candidates to be involved in residual feed intake. The findings of this study provided valuable resources to further clarify the genetic basis of regulation of feed efficiency in chicken from the perspective of lncRNAs.

## Introduction

Along with the increase in the world's population and the growing need for food, in particular, protein substances, meeting human nutritional needs is primarily an issue for human societies. Chicken meat is a high-quality protein source for humans due to the high levels of protein, low cholesterol and low calories. The poultry industry plays a vitally important role in converting grains into meat and eggs, which are essential sources of food for humans^1^. In response to the global demand for the chicken meat, genetic selection enabled us to improve many traits related to the functions of chickens and led to faster growth rates and higher feed efficiency in different commercial races such as Ross^2,3^. On the other hand, given the importance of native chicken breeds in rural economies in most of the developing and underdeveloped countries in having greater resistance to disease and the ability to tolerate harsh environmental conditions, their genetic improvement is reasonable in order to increase their efficiency. In this regard, Esfahan chicken is one of the native and dual-purpose breeds (to produce meat and eggs) in Iran^4^. Previous studies have demonstrated that feed efficiency is affected by many factors including food intake, genetic diversity in energy requirements for maintenance, the digestion, metabolism of nutrients, diversity in body composition and body temperature regulation^5^. Changes in the amount of energy required to digest food, the size of the digestive organs, and the amount of energy consumed within the tissues can all contribute to feeding intake variation^6^. Herein, birds with low-level RFI (Ross) means to consume less feed than predicted and are identified as efficient birds^7^. Furthermore, compared to the low efficient chickens, high efficient one may synthesize ATP more efficiently and control reactive oxygen species (Ross) production more strictly by enhancing the mitochondrial function in skeletal muscle^8^. According to the importance of Esfahan chickens compared to commercial breeds in having more resistance to diseases, efforts to improve chickens with efficient performance and a better feed efficiency would be beneficial with emphasis on genetic improvement. In this context, understanding the genetic mechanisms underlying the differences between native and commercial chicken breeds can be a promising strategy for a quick genetic improvement of the Esfahan breed.

Gene expression profiling is a powerful approach in functional genomics to uncover the complexity of cells and tissues at the transcriptional level. Next generation sequencing (NGS) based technologies are revolutionizing the field and paved the way to profile the gene expression of many organisms. In this context, RNA sequencing (RNA-Seq) is the current gold-standard method for genome-wide gene expression studies, which takes advantage of NGS. Hence, transcriptome analysis can help us to identify the molecular mechanisms affecting different traits such as feed efficiency. On the other hand, it is well known that the majority of the genome is expressed to non-coding RNAs (ncRNAs), which play critical roles in various biological processes^9^. One type of important ncRNAs that have been taken into consideration is Long noncoding RNA (lncRNA). LncRNAs are mostly defined as RNAs greater than 200 nucleotides in length, without any coding potential^10^. Most of the lncRNAs are processed like mRNA to be 5′ capped and 3′ poly adenylated as well as to be alternatively spliced^11,12^. In spite of lacking protein-coding potential, recent studies have shown the presence of short open reading frames (ORFs) that encode small peptides with vital biological functions. It has been reported that lncRNAs are less evolutionary conserved and tend to be expressed lower than that of protein-coding genes, as most of them are tissue specific. The most important functions of lncRNAs are regulating gene expression (by cis or trans-acting) through epigenetic, transcriptional, post-transcriptional, or post-translational mechanisms^11,13,14^.

A growing number of reports have demonstrated regulatory functions of lncRNAs in different chicken tissues using RNA-Seq. For instance, Li et al., identified differentially expressed lncRNAs from chicken skeletal muscle and reported that these genes might play important roles in the muscle development^15^. In a recent study, Muret et al., for the first time, reported a new lncRNA (lnc_DHCR24) as a key enzyme of cholesterol biosynthesis in chicken lipid metabolism^16^. Also, a study on six Beijing-you cocks, divergent in sperm motility led to the identification of 2,597 lncRNAs, including 1,267 intergenic lncRNAs (lincRNAs), 975 anti-sense lncRNAs and 355 intronic lncRNAs (ilncRNAs). Of these, 124 were differentially expressed and two lncRNAs including MSTRG.3652 and MSTRG.4081 and their target genes, showed their potential in sperm motility regulation^17^. A recent genome-wide study identified 9,393 tissue-specific lncRNAs (including 5288 novel genes) in eight tissues of chicken^18^. Moreover, 38 long intergenic non-coding RNA (lincRNAs) were reported in the duodenal transcriptome architecture of extreme RFI phenotypes in the six brown-egg dwarf hens^19^. In addition, many studies have suggested that lncRNAs play an important controlling role during lipid metabolism^16,20^, carbohydrate metabolism^21,22^, growth traits^23^, liver development^16,24^, ion transfer^25,26^ and digestive enzymes^23^ in chickens, which can affect feed efficiency in this way. In spite of abundant researches on feed efficiency of another farm animals^27,28^. limited researches have been conducted on the regulatory roles of lncRNAs in feed efficiency of chicken, especially in native breeds. Therefore, more studies that adopt genome-wide approaches are required to gain greater insight into these mechanisms. Additionally, there is a lack of information concerning the differences between native and commercial chicken breeds. In this study, RNA-Seq technology was applied to compare expressed lncRNAs in the liver tissue of two chicken breeds (Ross as a commercial and Esfahan as a native breed). The main goal was to gain a better understanding of biological and molecular mechanisms that are involved in the feed efficiency between native and commercial chicken breeds and establishing a foundation for future molecular studies. Moreover, using a stringent bioinformatic pipeline, novel lncRNAs were predicted, which can improve the annotation of the chicken genome.

## Material and methods

### Animals, RNA extraction and sequencing

RNA-Seq raw data used in this study was obtained from our previous study. Detailed information about the samples and the experimental design has been described previously^29^. Briefly, liver tissues of the chickens (including six Ross 708 chickens as a commercial breed and six Esfahani chickens as a native breed) were collected after the chickens were slaughtered. The chickens of both groups were raised in a birdhouse located in Safiabad Agricultural and Natural Resources Research and Education Center under the same environmental (temperature, humidity, and light) and dietary conditions with ad libitum access to the diet and water, for 42 days. To better understand the difference in the growth and feeding characteristics feed intake DFI and BWG of each individual were monitored on a daily basis from 24 to 42 d of age. All tissue samples were snap frozen in liquid nitrogen and then transferred to a − 80 °C freezer until required for RNA isolation. Total RNA was extracted from the tissue samples using TRIzol reagent (Invitrogen, Carlsbad, CA) based on the manufacture’s protocol. Three RNA samples from the same breed were pooled (by mixing together equal quantities of RNA) to generate a total of four pooled RNA samples (two samples in each breed) and were sent to BGI company (Shenzhen, China). Samples were applied for sequencing if the RNA Integrity Number (RIN) was > 7, based on an Agilent Bio Analyzer 2100 system. All the samples were subjected to a paired-end sequencing of 150 bp on the Illumina HiSeq 2000 platform (Illumina, San Diego, California, USA). The raw RNA-Seq data were deposited and released in SRA database, with the BioProject accession number of PRJNA707148 (https://www.ncbi.nlm.nih.gov/bioproject/PRJNA707148).

### RNA-seq data analysis

**Figure 1:**
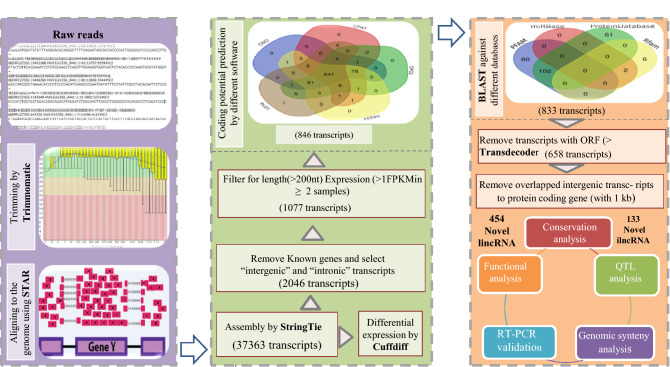
The bioinformatics pipeline for identifying annotated, known and novel lncRNAs. The middle and right Venn diagrams illustrate the results of the potential coding ability of the transcript using five software and blasting the transcripts against four different databases, respectively.

A computational approach using different filter criteria was applied to identify the lncRNAs (Fig. 1). Preliminary quality control analysis of the raw reads was checked with FastQC (v 0.11.2) (http://www.bioinformatics.babraham.ac.uk/projects/fastqc/)^30^. Raw reads were processed for initial trimming and filtering of the low-quality reads using the Trimmomatic tool (v 0.35)^31^. Then, STAR software (version 2.5.3.e) was applied to align the clean reads to the reference genome (Galgal5) using the default parameters^32^. StrnigTie tool (version 1.3.4.d) was used to assemble the aligned reads for each sample into transcripts^33^. Then, all the assembled transcripts of four samples were merged using the StrnigTie tool to generate a global and unified set of transcripts across the samples. This approach maximizes the overall quality of the final assembly. Differential expression analysis of the annotated genes was performed using Cuffdiff tool (v.2.2.1)^34^. To improve differential expression analysis for less abundant transcripts, the upper quartile normalization option was chosen. Finally, genes with a false discovery rate (FDR) ≤ 0.05 were considered as differentially expressed genes (DEGs) between the two breeds^34^. The reference genome for chicken and the annotation GFT file (release 91) were downloaded from the Ensemble database (https://www.ensembl.org/index.html)^33^.

### LncRNA Identification Pipeline

In order to discriminate the novel and known transcripts, the unified set of transcripts was compared against the Ensemble chicken genome annotation (release 91) by Gffcompare (https://github.com/gpertea/gffcompare). Using this approach annotated lncRNAs were found. Then, only the unknown intergenic (marked with class code “u”) and intronic (marked with class code “i”) transcripts were subjected to further analyses. All unknown transcripts were blasted against NONECODE database (v5.0) (http://www.noncode.org) to identify the reported chicken lncRNAs in this database. It is worth noting that there are a large number of chicken lncRNAs in this database that are not included in the Ensemble chicken genome annotation. All the significant hits (E-value = 1e−5) were considered as known lncRNAs and were further classified to intergenic lncRNAs (lincRNAs) and intronic lncRNAs (ilncRNAs) based on their class codes. Different kinds of lncRNAs based on their genomic origins relative to nearby protein-coding genes can be grouped as sense or antisense transcript overlapping and as promoter associated, intronic or intergenic^35^. An ilncRNA possesses a transcribed region overlapped with the intron segments of protein-coding genes, and a lincRNA is located in the intergenic region lacking known protein-coding genes^36^. Also, lincRNAs have high relative abundance in the genome, for instance^37^. Kuo et al. in their study found that lincRNAs accounted for more than half of chicken lncRNAs. For the remaining transcripts, longer than 200 nt, the following stringent filtering criteria were performed to identify the potential novel lncRNAs^37^. First, the transcripts with one exon that were longer than 10,000 nt or those were located in the genomic regions containing simple sequence repeats (based on UCSC RepeatMasker file) were removed. Second, the transcripts with FPKM ≥ 1 expressed in at least two samples were kept. Third, all the remaining transcripts that were blasted (E-value < 1e-5) against one of the following databases were excluded from further analysis: UniprotKB (https://www.uniprot.org/help/uniprotkb) (by BLASTx), miRbase (https://www.mirbase.org/) (release 21, by BLASTn) and Rfam (http://rfam.xfam.org/) (by BLASTn)^38^. Also, Hmmscan tool from the HMMER package (version 3.1b2) was applied to evaluate which of the remaining transcripts contains a known protein domain with any of the known protein family domains in the Pfam database^39^. The significant hits were then removed. Forth, to predict the coding potential of the remaining transcripts, five software including CPC2 (version 2 beta) (score > 0.5)^40^, CNCI (version 2) (score > 0)^41^, CPAT (version 1.2.4) (score > 0.36)^42^, PLEK (version 1.2) (score > 0)^43^ and FEElnc (version 12/07/2016) (default parameters)^44^ were used. Only the transcripts that were simultaneously considered to have coding potential by at least three of the mentioned tools were excluded from further analysis. Next, putative protein-coding transcripts were filtered out by considering open reading frame (ORF) length greater than 300 aa, which was predicted using TransDecoder tool (v2.1.0) (https://transdecoder.github.io/). In the last step, the intergenic transcripts that were located in a distance < 1 kb to a known protein-coding gene, were excluded. Finally, all the transcripts that successfully passed the filtering steps were considered as putative novel lncRNAs and classified into lincRNAs and ilncRNAs classes (Fig. 1).

### Functional analysis

To investigate various aspects of the putative lncRNAs, five different analyses were performed based on our previous study, which is described as follow^38^:

1. **Analysis of conservation** Previous studies have shown that lncRNAs are not well conserved at the sequence level^45,46^. To assess this, all the putative novel lncRNAs were blasted against lncRNA sequences of human, rat and cow using BLASTn^38^. All the lncRNA sequences were obtained from NONCODE database (v5.0). The hits meeting the E-value threshold < 10–5 were considered significant.
2. **Genomic synteny analysis** It is well known that establishing homology relationships among lncRNAs is challenging due to their low sequence conservation. Previous studies suggested that genomic position (synteny) of lncRNAs is more conserved than their cross-species sequence conservation. Therefore, lncRNAs conservation is geared toward the maintenance of synteny^47^. In other words, suppose neighboring upstream and downstream protein coding genes of a lincRNA in chicken are X and Y. If there be a lincRNA in other organisms that is located between the same X and Y protein coding genes, it can be considered as a conserved syntenic lincRNA. Here, to identify syntenic lncRNAs, order of surrounding genes flanking each putative novel lincRNA was compared for conserved synteny in the human and bovine genomes. All the genomic positions of lincRNAs were extracted from the NONCODE database (v5.0) for human and bovine.
3. **Target prediction and functional enrichment analysis** Typically, lncRNAs regulate their target genes through cis or trans modes. The cis-acting refers to the regulation of genes in a host (in the case of ilncRNAs) or nearest neighboring protein coding genes (in the case of lincRNAs). According to the location of the lncRNAs and mRNAs on the chicken genome, 10/100 kb upstream or downstream of the differentially expressed lncRNAs were searched to identify the nearest neighboring protein coding genes, which can be considered as potential target genes of these lincRNAs^20^. On the other hand, trans-acting refers to the influence of lncRNAs on other genes at expression levels, which can be assessed by co-expression analysis (correlating expression levels between lncRNAs and mRNAs)^11,14^. To predict the potential target genes of the lncRNAs in trans mode, Pearson correlation coefficient was calculated based on the expression levels of each pair of lncRNA and mRNA (r > 0.95 or r <  − 0.95). Functional enrichment analysis including Gene Ontology (GO) and KEGG pathway analysis^48^ related to the predicted target genes of lncRNAs was conducted using the EnrichR database^49^. A term with an adjusted P-value < 0.1 (FDR) was considered to be significantly enriched.
4. **QTL mapping analysis of LncRNAs** The co-localization analysis was performed to determine if the putative novel lncRNAs are located in the QTLs related to feed efficiency. For this purpose, all QTLs related to feeding efficiency were retrieved from AnimalQTLdb^50^. Then positions of the lncRNAs were compared to the QTL's locations.
5. **Target prediction of the candidate LncRNAs and PPI network** The potential protein–protein interaction (PPI) network was constructed for the predicted cis and trans target genes of the lincRNAs and ilncRNAs (known and novel) using STRING database (Search Tool for the Retrieval of Interacting Genes/Proteins, https://string-db.org/). ^51^Then, Cytoscape software (version 3.7)^52^ was applied to visualize and combine the significant interactions obtained by STRING along with predicted cis and trans target genes of lncRNAs (separately for each of lincRNAs and ilncRNAs). The key sub-networks (module genes) were detected using a Cytoscape plugin, ClusterONE tool (version 1.0), based on minimum size = 5 and a minimum number of the genes in a module = 5^53^. In order to further explore the characteristics of the significant modules, functional enrichment analysis was performed on their gene members.

### Quantitative real-time PCR (RT-qPCR) validation

To validate the RNA-Seq results by RT-qPCR, seven differentially expressed lncRNAs (including four known lincRNAs and three novel lincRNAs) were randomly selected. Two biological replicates were conducted for each breed. Total RNA was extracted from the liver of chickens using TRIzol (Invitrogen, CA, USA) according to the manufacturers’ protocols and the concentration and purity of RNAs were measured using NanoDrop 2000. The cDNA was synthesized from purified RNA by using an RT-qPCR kit (Takara, Dalian, China). Primer 3 plus software was applied to design the primers^54^. The details of all the designed primers are provided in Supplementary File S1. RT-qPCR was performed on a Light Cycler 96 instrument (Roche Co. Germany) using HiFi SYBR Green Master Mix (Thermo Fisher Scientific, USA). RT-qPCR was performed with the following thermo cycling conditions: an initial 1 cycle at 95 °C for 10 min, 40 cycles at 95 °C for 15 s, 60 °C for 20 s and 72 °C for 20 s, followed by a 72 °C elongation for 60 s. GAPDH and ACTB were used as house-keeping genes to normalize the expressions of lncRNAs^55^. The results were analyzed using the 2 − ΔCt method.

### Ethics approval

All methods were carried out in accordance with relevant guidelines and regulations. All experimental protocols were approved by the Animal Care and Use Committee at the Animal Science Research Department, Safiabad Agricultural.

## Results

### Phenotype measurements

In our previous study, the average BWG of higher RFI and lower RFI groups had obtained 441.06 ± 6.76 g and 1027.88 ± 7.57 g, respectively. The significant differences (p < 0.01) in daily feed intake (DFI) had observed between native and commercial chickens (1329.25 ± 8.05 vs 2007.90 ± 9.25). The lower RFI had shown a higher average daily weight gain than a higher one. Consequently, the differences in mean RFI values between LRFI and HRFI chickens in each breed were highly significant (p < 0.01). The local breed had the RFI values of 13.430 ± 5.393 (g/day) compared with –11.212 ± 4/435 (g/day) for the commercial breed during 19 days (day 24–42) of the experiment".

### RNA-seq data analysis

A total of 35,696,493 and 34,824,326 raw paired-end reads were obtained in native and commercial breeds, respectively. Following a pipeline of adaptor removal, quality filtering and the removal of sequences that were too short, only 565,714 and 534,690 paired reads did not pass the quality filtering and were removed in native and commercial breeds, respectively. In total, 84.70–86.97% of the clean reads from all the samples were successfully aligned to the reference genome. Of the successfully aligned reads, 82.90–84.53% were uniquely aligned (Table 1).

**Table 1:** Number of RNA-Seq reads and mapping rates of all samples.

Sample name	Raw reads	Clean reads	Uniquely mapped reads (%)	Total mapped reads (%)
Native 1	14,510,042	14,246,264	11,810,777 (82.90)	12,140,023 (85.22)
Native 2	21,186,451	20,884,515	17,653,576 (84.53)	18,162,985 (86.97)
Commercial 1	19,034,129	18,723,445	15,607,073 (83.35)	16,028,736(85.61)
Commercial 2	15,790,197	15,566,191	12,859,795 (82.61)	13,183,626(84.70)

### LncRNA identification and characterization

To comprehensively identify lncRNAs in the genome wide, a stringent bioinformatics pipeline was applied. In total, 37,663 transcripts were generated through reconstructing the transcripts of all the samples by StringTie. All assembled transcripts were compared against ENSEMBL chicken GTF file and 20,846 and 1,110 transcripts were annotated as mRNAs and lncRNA, respectively. After comparing the remaining transcripts against chicken lncRNAs from the NONCODE database, 593 transcripts were identified as known lncRNAs (including 525 lincRNA ad 68 ilncRNAs), which are not annotated in the chicken GTF file (ENSEMBL database), yet. Finally, according to our stringent lncRNA prediction pipeline, 587 putative novel lncRNAs including 454 lincRNAs and 133 ilncRNAs were identified (Fig. 1).

To understand the differences in characteristics of novel lncRNAs and verify their accuracy, their exon number, GC content, transcript length and expression level were compared against known and annotated lncRNAs as well as mRNAs.

**Figure 2:**
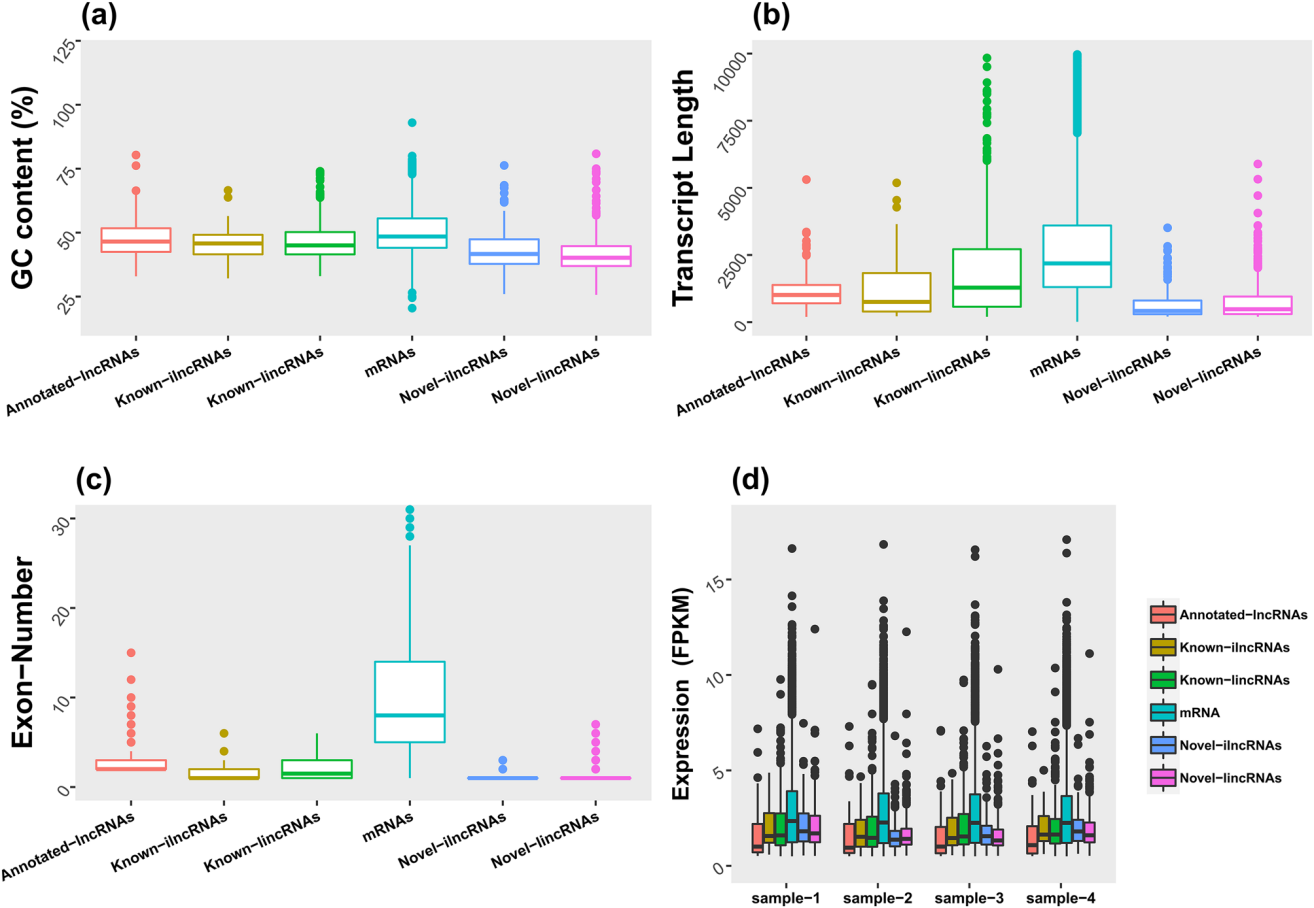
Comparison of (**a**) GC contents, (**b**) transcript length, (**c**) exon number and (**d**) gene expression levels of the annotated, novel and known lncRNAs with mRNAs.

The results revealed that the average GC content of the novel lincRNAs and novel ilncRNAs was similar (approximately 42%), which is lower than known mRNAs (50%) (Fig. 2A). GC content of annotated lncRNAs, known ilncRNAs, known lincRNAs, novel ilncRNAs and novel lincRNAs were 47, 46, 47, 43 and 42%, on average, respectively. The transcript lengths of novel lncRNAs ranged from 203 to 5889 bp, with an average of 733 bp, which was lower than the values observed for mRNAs (with average length 3193.793 bp) (Fig. 2B). The average transcript length of annotated lncRNAs, known ilncRNAs, known lincRNAs, novel ilncRNAs and novel lincRNAs were 1,100, 1,617, 1,995, 685, and 782, respectively. On average, the exon numbers associated with novel lncRNAs (1.18 on average) were clearly less than mRNAs (10.82 on average) (Fig. 2C). Exon numbers of annotated lncRNAs, known ilncRNAs, known lincRNAs, novel ilncRNAs and novel lincRNAs were 2.8, 1.6, 2.6, 1.14 and 1.3, on average, respectively. Most novel lncRNAs (571 of 587, 97%) contained no more than two exons, while above 83% mRNAs had no less than three exons. The expression levels of mRNAs and novel lncRNAs were further compared according to FPKM values, and the Fig. 2D shows that the expression levels of mRNAs in all samples were higher than lncRNAs (the average expression of annotated lncRNAs, known ilncRNAs, known lincRNAs, mRNAs, novel ilncRNAs and novel lincRNAs in different samples were 0.39, 1.41, 1.56, 1.67, 1.53, and 1.46, respectively).

**Figure 3:**
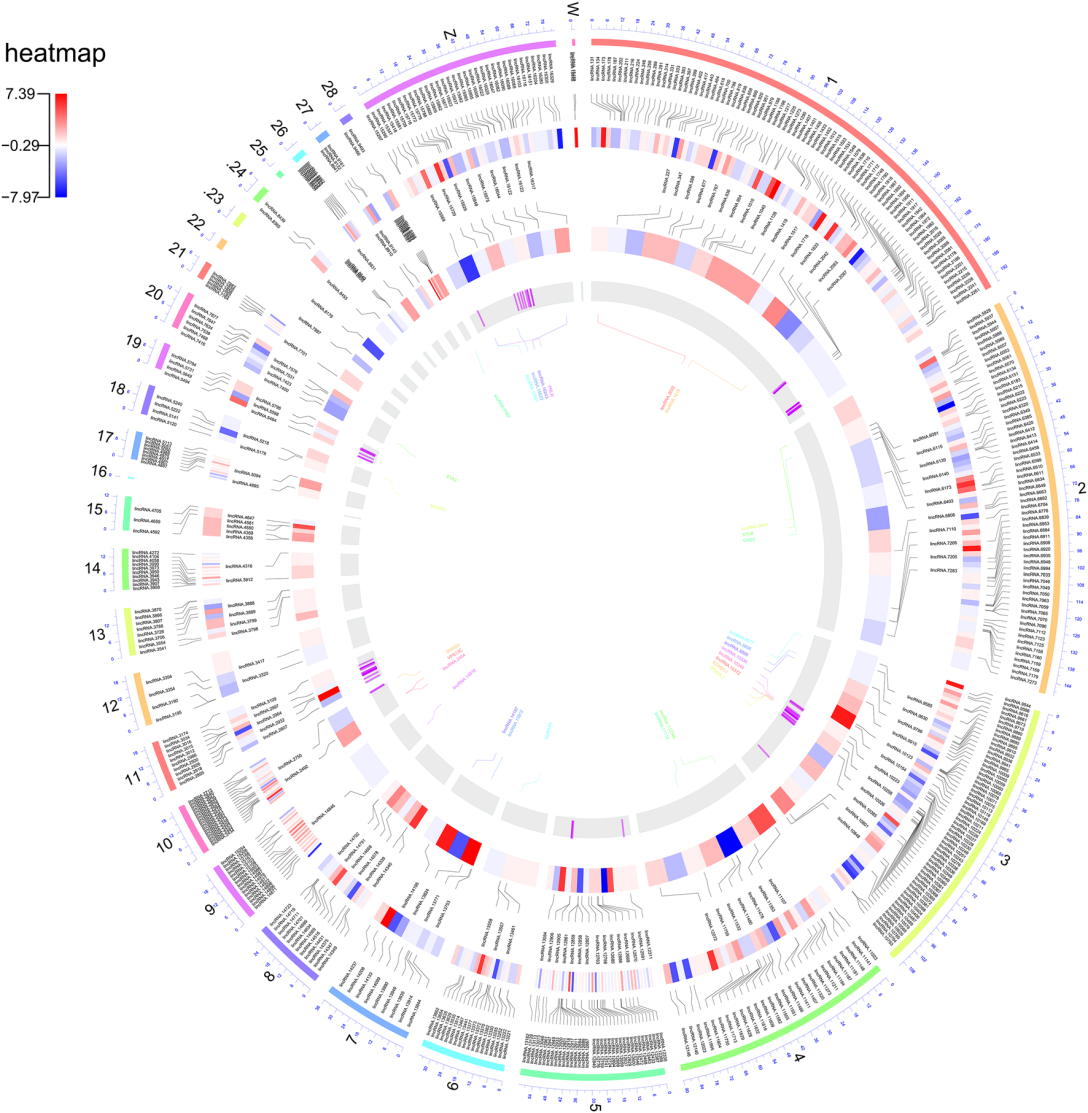
Circos plot represents the genome-wide distribution density of all the identified novel lncRNAs (in clockwise order). From outside to inside the plot shows the chromosomes, and the next ring the position of the novel lincRNAs, the log2 fold change (Esfahani against Ross) of the novel lincRNAs, position of the novel ilncRNAs, the log2 fold change (Esfahani against Ross) of the novel ilncRNAs, the location of Residual feed intake QTLs from animal QTL database and the names of promising lncRNAs and mRNAs pairs associate with Residual feed intake. Red heatmap colors represent the higher expression of the gene in Esfahan than Ross breed.

In terms of chromosomal distribution, the novel lncRNAs were distributed across nearly all of the chicken chromosomes, except chromosome 16. Chromosome 1 has the greatest number of lncRNAs (97 lncRNAs), followed by chromosome 2 (72 lncRNAs), 3 (71 lncRNAs), 5 (51 lncRNAs) and Z (43 lncRNAs), whereas chromosomes 22, 23 and 25 contained only ilncRNAs (1, 1 and 3), respectively (Fig. 3).

### Conservation analysis

Out of 454 novel lincRNAs, six, nine and 12 lincRNAs were found to have homology to bovine, mouse and human lincRNAs, respectively. Also, three, five and four known lincRNAs had conserved sequences in bovine, mouse and human, respectively. Moreover, two and two novel ilncRNAs and one and two known ilncRNAs were evolutionary comparable with lncRNA sequences from bovine and mouse, respectively. No significant hit was found in the comparison of ilncRNAs (known and novel) and human lncRNAs. In addition, of 16 novel and nine known conserved lincRNA transcripts, eight and two genes showed evidence of sequence homology among bovine, human or mouse (Supplementary File S2). Overall, transcript-level homology of our predicted lncRNAs with active transcribed lncRNAs in human, cow and mouse detected, and found that only 2% of known and 3% of novel lncRNAs can be aligned to investigated species. In agreement with our results, it was reported that less than 1% of chicken lincRNAs owned detectable sequence conservation with human or mouse lincRNAs^17^.

### Synteny analysis

The results of the synteny analysis indicated a similar structural architecture between the performed comparisons. Results of this analysis revealed that 62 known and 96 novel lincRNAs as well as 89 known and 116 novel lincRNAs were located in the neighborhood of respective orthologous protein coding genes in chicken/cow and chicken/human comparisons, respectively (Supplementary File S3). Out of 62 and 96 lincRNAs, two and three lincRNAs had conserved sequencing with bovine and human, respectively. Also, two known and four novel lincRNAs, out of 89 and 116 syntenic genes, had an orthologous sequence in bovine and human, respectively.

Moreover, 121 known lincRNAs and 212 novel lincRNAs were determined with conserved synteny among the investigated species. Of these, 26% (31 of 121) of known lincRNAs and 23% (48 of 212) of novel lincRNAs showed sequence homology with the investigated species. These findings were consistent with the previous study that reported higher conservation in synteny of the lncRNAs in comparison to their sequences^38^.

### Differential expression analysis

In total, 17,833 transcripts (including 398 annotated transcript lncRNAs, 10,624 known genes and 6,810 novel transcripts) were identified to be expressed in both breeds. Of these, 1,040 and 1,081 transcripts were expressed only in commercial and native breeds, respectively. Therefore, 15,712 transcripts were commonly expressed in both breeds. To identify potential feed efficiency related genes, differential expression analysis was applied and FPKM values of the genes were compared between the two chicken breeds. The analysis showed that 39 known lncRNAs (including one up-regulated ilncRNAs, eight up and 30 down-regulated lincRNAs) and 87 known mRNAs (including 63 up- and 24 down-regulated) were DEGs. In addition, 14 novel lincRNAs (four up and 10 down-regulated) tended to be differentially expressed. No annotated lncRNA and novel ilncRNAs were identified to be differentially expressed. The top five differentially expressed lncRNAs in each lncRNA class are represented in Table 2 (the complete list of these genes is provided in Supplementary File S4).

**Table 2:** The top five differentially expressed genes lncRNAs classes between the two breeds and their cis target gene.

Source	ID	Closest left mRNA	Closest right mRNA	Expression in native Esfahan	Expression in commercial chicken	Adjusted p-value
Novel lincRNA	lincRNA.10349.1	KIAA0408	ECHDC1	2.86443	0	5.00E−05
	lincRNA12629.1	RTF1	ENSGALG00000008599	3.55402	17.0844	5.00E−05
	lincRNA.224.1	FAM19A5	ENSGALG00000039992	5159.49	1740.5	5.00E−05
	lincRNA15581.1	PRLR	SPEF2	0	1.87716	0.00015
	lincRNA.5050.1	TRAF1	C5	1.48732	4.27511	0.00065
Known lincRNA	lincRNA.1424.2	SH3BGR	B3GALT5	5.90444	15.7968	5.00E−05
	lincRNA.16016.1	RNF38	TRIM14	17.4264	6.9754	5.00E−05
	lincRNA.14534.1	LRRC8D	LRRC8C	4.24643	11.6967	5.00E−05
	lincRNA.3640.1	SLC26A2	ENSGALG00000001206	65.8459	32.8307	5.00E−05
	lincRNA.7295.1	COL22A1	KCNK9	7.77861	34.7036	0.00025
Known ilncRNA	ilncRNA.8900.7	ENSGALG00000043654	ENSGALG00000043654	21.2565	9.19081	0.00035

### RNA-seq data validation by RT-qPCR

**Figure 4:**
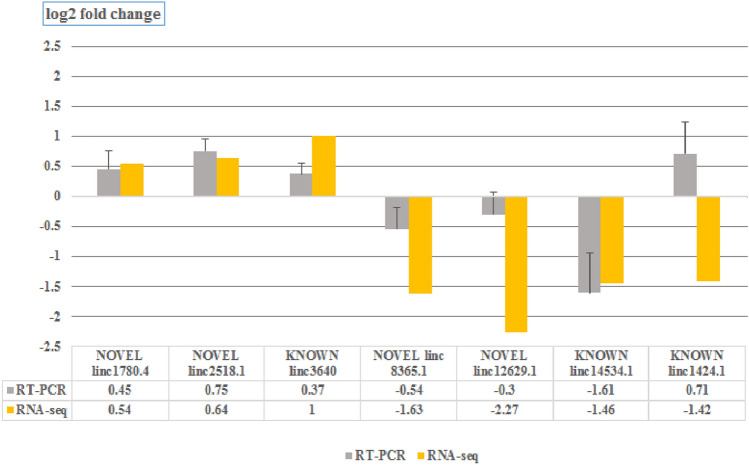
Validation of seven randomly selected novel lincRNAs by RT-qPCR. The fold change represents the ratio of average expression of native samples relative to that of commercial breed**.**

To further confirm the accuracy of the sequencing data, RT-qPCR was performed on seven differentially expressed lincRNAs. As shown in Fig. 4 a similar expression pattern (except linc.1424.1) was observed in RT-qPCR compared to RNA-Seq data. However, there were variations observed in these methods, which can be attributed to intrinsic features of these approaches.

### LncRNA target genes and functional analysis

To understand the biological functions of the lncRNAs, cis-regulated genes of these genes were investigated. The genes transcribed in the 10/l00 kb upstream or downstream around the lincRNAs are generally considered to be cis target genes. All 52 (38 known and 14 novel) differentially expressed lincRNAs were detected to be located near of 70 mRNAs and these mRNAs were involved in "lipid metabolism", "carbohydrate metabolism", "growth" and "cell death".

Trans target genes of the known and novel lncRNAs were predicted through expression correlation analysis. In total, 53 lincRNAs (48 known and five novel lincRNAs) were corresponded to 619 target genes (Supplementary File S5). The identified co-expressed genes were similar for the most of known lincRNAs. Accordingly, lincRNAs with similar target genes were clustered, which led to four clusters. The first, second, third, and fourth clusters included 4, 3, 15, and 24 lincRNAs, which were co-expressed with 179, 170, 133, and 130 target genes, respectively. Functional enrichment analysis of the target genes in cluster number one and two were significantly enriched in one and three GO terms and one and three KEGG pathways, respectively, which were related to biological processes such as “gamma-aminobutyric acid signaling pathway”, “ear morphogenesis”, “cochlea morphogenesis” and “inner ear morphogenesis”. Also, nine and 29 significant GO terms were found in clusters number three and four, respectively. The detailed information related to these clusters and their functional analysis are provided in Supplementary File S6. According to the previous studies, feed efficiency-related terms in clusters number three and four were detected including “monocarboxylic acid transport”, “potassium ion transport”, “chloride ion homeostasis”, “negative regulation of calcium ion trans membrane transport” and “cellular response to cAMP”. KEGG pathway analysis was further performed and no significant pathways were identified.

**Figure 5:**
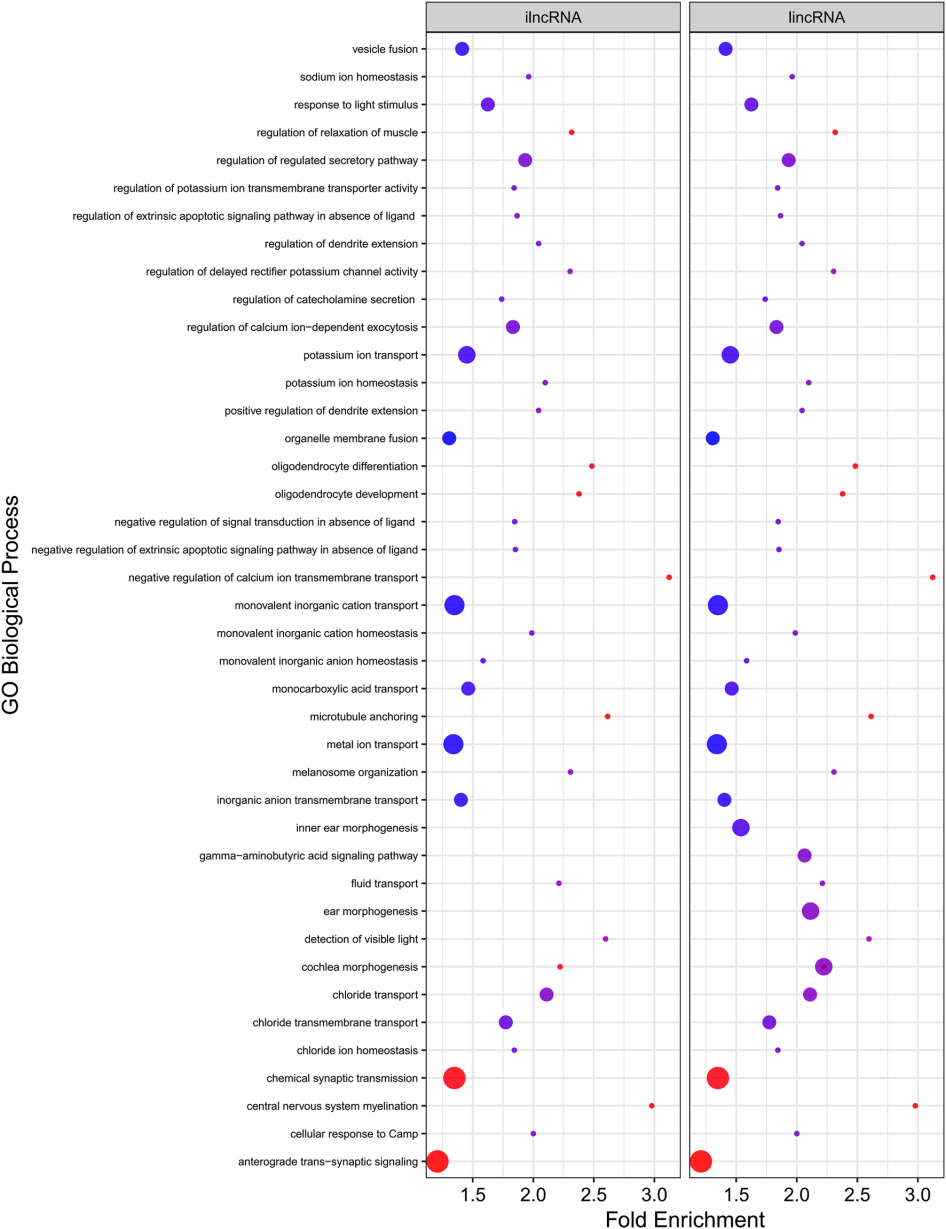
Functional enrichment analysis of the trans target genes of predicted known lincRNAs and ilncRNAs.

In ilncRNAs, 10 known ilncRNAs were corresponded to 265 target genes (Supplementary File S5). Based on the co-expressed genes of the known ilncRNAs, which were similar among the ilncRNAs, two clusters were constructed. Three ilncRNAs were expressed in cluster number one with 130 target genes. Also, there were four ilncRNAs corresponding to 130 target genes in the second cluster. Interestingly, GO analysis of these target genes led to detection of 38 enriched terms (nine GO terms in cluster one and 29 GO terms in cluster two) (Fig. 5), which were similar to those observed in clusters number three and four of lincRNAs (Supplementary File S6).

**Figure 6:**
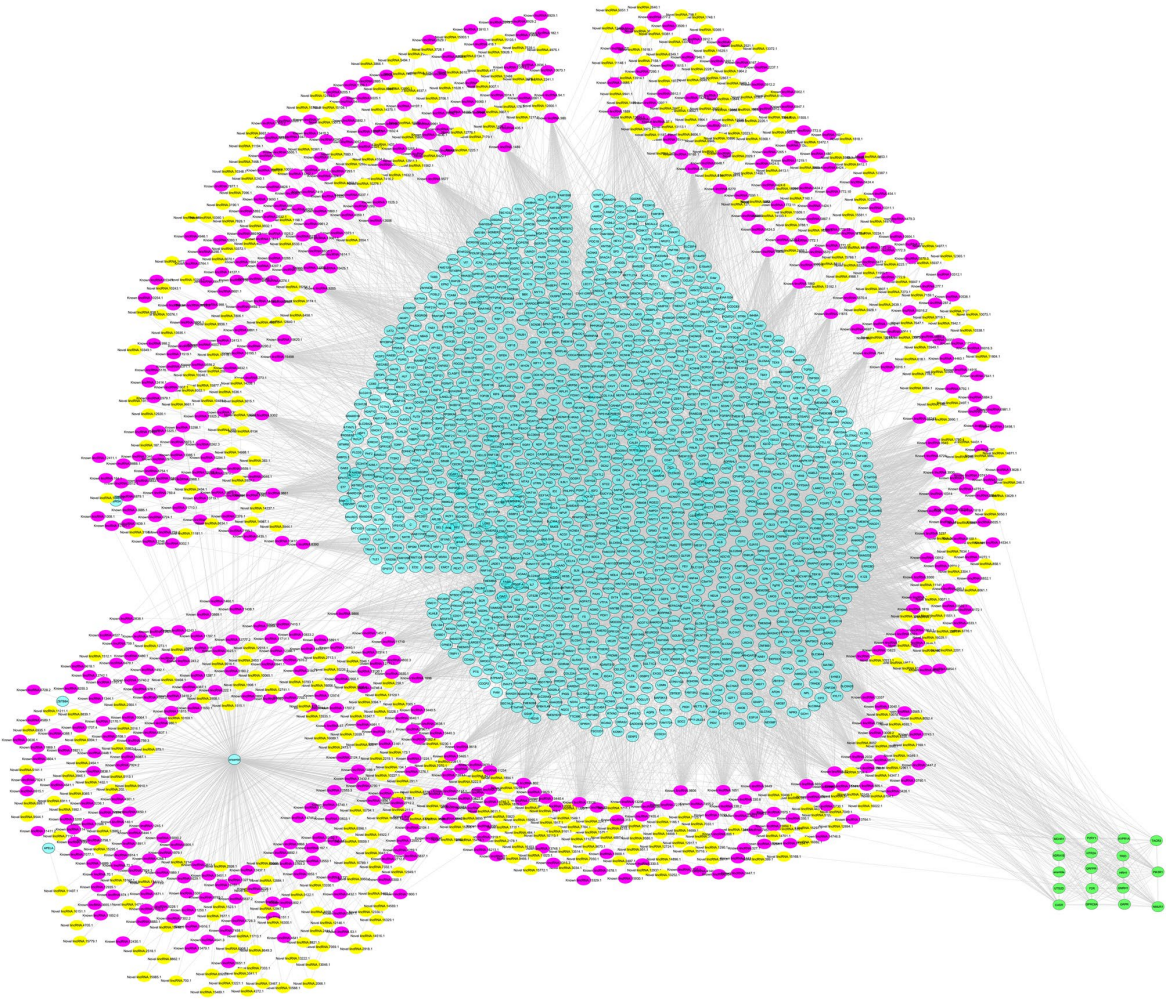
Final constructed network based on the known and novel lincRNAs. Purple nodes: Known lincRNAs, yellow nodes: Novel lincRNAs, Sky blue nodes: protein coding genes, green nodes: predicted module (P-value = 0.007).

**Figure 7:**
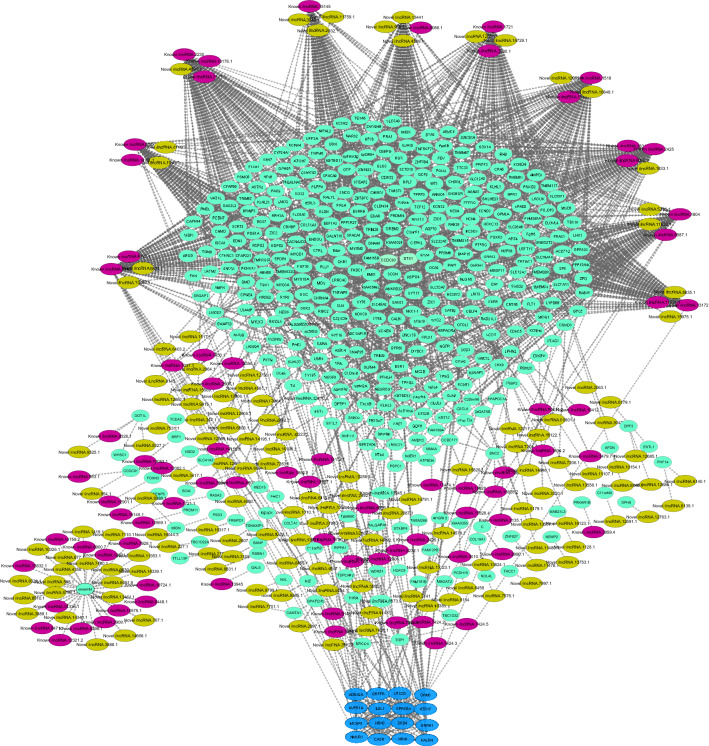
Final constructed network based on the known and novel ilncRNAs. Purple nodes: Known ilncRNAs, Light Olive nodes: Novel ilncRNAs, Medium Spring Green nodes: protein coding genes, light-blue nodes: predicted module (P-value = 6.147-e7).

Next, PPI network construction was performed based on the target genes of lncRNAs. In total, two significant PPI networks were obtained including 1,320 and 398 genes and 5,129 and 440 interactions for lincRNAs and ilncRNAs, respectively. After combining PPI network and cis and trans target genes of lincRNAs, 1,029 lincRNAs (573 known and 454 novel) were connected to 1,514 mRNAs with 11,160 connection edges. Moreover, a network with 216 ilncRNAs (68 known and 168 novel) and 416 protein-coding genes along with 1,490 interactions were obtained. ClusterONE tool predicted one significant module in each of the networks with < 20 nodes including green module (P-value = 0.0007, Fig. 6) in lincRNA-based network and light-blue module (P-value = 6.147Ǝ-7, Fig. 7) in ilncRNA-based network.

Functional enrichment analysis indicated that genes in green module (lincRNA-based network) were significantly enriched in 165 GO terms and seven KEGG pathways, which were related to glucose homeostasis (GO:0042593), calcium ion transport (GO:0006816), insulin receptor signaling pathway (GO:0008286), lipid phosphorylation (GO:0046834). The light-blue module (ilncRNA-based network) were enriched in 132 metabolic pathways and four KEGG pathways including “positive regulation of ion transport”, “positive regulation of sodium ion trans membrane transporter activity”, “negative regulation of lipid metabolic process” and “carbohydrate homeostasis”. The functional enrichment analysis of the two modules showed that both are related to feed efficiency (Supplementary File S7).

### QTL analysis

To further investigate the potential of the lncRNAs in feed efficiency related traits, a co-localization analysis was performed with feed efficiency associated QTLs. The current version of the chicken QTLdb includes 11,340 QTLs representing 347 different traits. Of these, eight hundred and thirty-four QTLs that were associated with six traits related to feed efficiency were considered. Of these, 29 QTLs were found to be overlapped with the novel lncRNAs. In this regard, out of 454 and 133 novel lincRNAs and ilncRNAs, 47 and 11 lncRNAs were located in 19 and 10 QTLs, respectively, including 36 lncRNAs in “feed intake” and 23 lncRNAs in “residual feed intake” (Supplementary File S8). Of these, ilncRNA.2932 and lincRNA.5494, which were located in feed intake related QTLs, were specifically expressed in commercial breed. Out of 47 novel lincRNAs, one differentially expressed lincRNA (lincRNA.10349) was overlapped with a QTL related to residual feed intake (RFI). Moreover, seven lincRNAs (lincRNA.1964, lincRNA.2201, lincRNA.2215, lincRNA.5649, lincRNA.2920, lincRNA.2178, and lincRNA.2186) were located in more than one QTL related to feed efficiency (Supplementary File S8). Interestingly, several cis target genes of these lincRNAs, including KCNRG, DLEU7, CTSC and RAB30, were reported to be involved in feed efficiency and RFI or has a known role the growth rate ^56–58^. These genes can be considered as promising candidate genes responsible for feed efficiency in chicken (Table 3).

**Table 3:** The list of identified novel lincRNAs in more than one QTL related to feed efficiency.

lncRNA	lncRNA position		Number of QTL	QTL Id		Closest left mRNA	Closest right mRNA
lincRNA.2178	1:186,194,986,186,196,686		3	64,551,64,552,64,553		SLC36A4	–
lincRNA.1964	1:169,421,116–169,445,490		3	64,842,64,815, 64,927		KCNRG	DLEU7
lincRNA.2186	1:186,422,650- 186,423,467		3	64,551, 64,552, 64,553		–	FAT3
lincRNA.2201	1:188,331,186- 188,331,518		3	64,551, 64,552, 64,553		CTSC	RAB38
lincRNA.2215	1:189,167,401–189,168,015		3	64,552, 64,553, 64,555		RAB30	–
lincRNA.2920	11: 6,752,337–6,753,098		2	64,559, 64,560		PAPD5	HEATR3
lincRNA.5494	19: 6,298,902–6,299,353		2	64,567, 64,568		GOSR1	ABR

## Discussion

Recently, lncRNAs have received much consideration and a growing number of studies have shown that lncRNAs play key roles in various physiological and pathological processes^59,60^. Whereas many of the lncRNAs and their functions are known in different species, such as humans and mouse, lncRNA research is in its infancy in domestic animals, especially in chickens^61^. In addition, the role of lncRNAs in the liver of chicken in regulating feed efficiency-related traits is unclear. The most common way of identifying relevant lncRNAs is by differential expression between contrasting conditions^38^. Hence, several potential known and novel lncRNAs were identified in the present study by comparing the gene expression profile of the liver of two extremely different chicken breeds (Iranian native chicken vs Ross breed). Comparing the features of the identified novel lncRNAs to protein coding genes showed consistency between our results and the previous studies, as lncRNAs had a lower expression, shorter transcript length^62^, fewer exons^63^ and lower GC content relative to mRNAs^38,64^. These findings indicate that the used bioinformatic pipeline is reliable. Only 2% of known and 3% of novel lncRNAs showed transcript-level homology with known lncRNAs in human, cow and mouse was, which is in agreement with Kuo et al.^37^. In this regard, Liu et al. in their study reported that less than 1% of chicken lincRNAs owned detectable sequence conservation with human or mouse lincRNAs and confirmed that lncRNAs in vertebrates have low levels of interspecies short and highly conserved regions^17^. Also, in agreement with our previous study, synteny and conservation analysis of the novel lncRNAs emphasized that synteny of lncRNAs is more conserved than their cross-species sequence conservation^38^. Therefore, syntenic analysis can be considered as a useful approach for improving the prediction of novel lncRNAs in the genomes that are incompletely annotated. In brief, 1,110 annotated lncRNAs, 525 known lincRNAs, 68 known ilncRNAs, 454 novel lincRNAs and 133 novel ilncRNAs were found. Of these, 38, 1, and 14, known lincRNAs, known ilncRNAs and novel ilncRNAs, respectively, were identified as DEGs. Although differentially expressed genes can be considered as potential candidates related to feed efficiency, however further investigations are needed to be ensured about their potential roles in these biological processes. Hence, different functional analysis was applied to further understand the functions of these genes in regulating feed efficiency in chicken.

Recent studies suggested that the function of lncRNAs can be deduced by analyzing their co-expressed mRNAs or neighboring protein-coding genes in genome wide^65^. Accordingly, in the cis mode, the identified target genes of the novel lincRNAs were mainly involved in lipid metabolism (Lipolysis, cholesterol biosynthesis) and carbohydrate and growth metabolism, which are related to feed efficiency and make these novel genes as ideal candidates to investigate the regulatory mechanism of feed efficiency in chicken. The relationship between lipid metabolism and feed efficiency has been reported in previous studies, as animals with lower feed efficiency have higher fat deposition and cholesterol levels^66,67^. One of these target genes was ethyl malonyl‑CoA decarboxylase (ECHDC1, targeted by novel lincRNA.103491.1), which is a new metabolite proof reading enzyme. ECHDC1 can eliminate ethyl malonyl‑CoA by converting it to butyryl‑CoA^68^. Interestingly, ECHDC1 was a DEG in our study that was up-regulated (fold change = 0.52) in Ross breed and showed an opposite expression pattern with its neighboring lincRNA.103491.1. Moreover, these genes were located in the QTL regions associated with feed efficiency, which reinforce their potential function in this subject. These findings make these genes (lincRNA and its target gene) as interesting candidates for a follow-on experiment to assess their impact on feed efficiency in chicken. Lipolysis proceeds from the hydrolytic cleavage of ester bonds in triglycerides (TGs), and result in the generation of fatty acids (FAs) and glycerol^69^. Increased lipolysis of fatty tissues may increase circulating fatty acid concentrations and displacing the muscle's preference for fatty acid oxidation^70^. Moreover, excessive fat accumulation has a negative effect on feed efficiency^71^. VPS13C, a member of the VPS13 family of proteins (VPS13A, B, C, and D), regulates galectin-12 stability in adipocytes^72^, was predicted to be targeted by a novel lincRNA.2454.1 and its target gene was up regulated (fold change = 0.17) in high efficient chickens (commercial breed). In this regard, the important roles of galectin-12 in adipocyte differentiation and lipolysis for the regulation of whole-body adiposity have been reported^72^. In addition, VPS13C is associated with regulation of glucose homeostasis^72^. Lemley et al. in their study suggested this gene to play an important role in glucose homeostasis for high milk production in dairy cow^73^. Therefore, the glucose homeostasis process probably stimulated lipolysis process^74^, indicating that high efficient chickens have a lower amount of abdominal fat^75^.This finding also emphasized the functional diversity of lncRNAs, which may contribute to widespread regulatory roles in the liver tissue of chicken. The membrane-bound PRLR, as a member of the cytokine receptor family, is closely related to the growth hormone receptor. Over 300 separate biological activities have been attributed to PRL including endocrine signaling, metabolism, control of water and electrolyte balance, growth and development^76^. PRLR is predicted to be a target gene of lincRNA.15581.1 and its higher expression in commercial chicken (fold change = 0.98) can increase feed efficiency by increasing growth rate and decreasing storage energy^77^.

The genes that were identified as targets of differentially expressed known lincRNAs and also were associated with efficiency-related traits were included B3GALT5, ACAA2, CDCA7L, CHST7, LRRC8D, LRRC8C, LMAN1, CTDSPL, EBP, and AGPAT3. Some biological processes like lipid metabolism^45,75,78^ and cholesterol biosynthesis^66,79^, were known to be associated with feed efficiency. The ACAA2 gene that was predicted to be targeted by lincRNA.15325 implicated in mitochondrial fatty acid oxidation. ACAA2 encodes an enzyme that catalyzes the cleavage of 3-ketoacyl CoA to yield acetyl-CoA and acyl-CoA, the final step of the mitochondrial fatty acid beta-oxidation spiral. The high expression level of ACAA2 was found in the muscle of the high feed efficient birds^78^. Furthermore, a previous study by focusing on growth traits and important metabolic pathways in pigs reported ACAA2 as a candidate gene involved in fatty acid metabolic, which is an essential source for energy^80^. Accordingly, high efficient chicken had higher expression of genes involved in fatty acid uptake and oxidation^78^. The up regulation of lincRNA.15325 and its related target gene (fold change = 0.13) in the commercial breed may lead to higher fatty acid metabolism to supply energy. Cholesterol is an essential component of animal cell membranes that gives cell membranes elasticity and fluidity and is involved in cellular signaling and intracellular transport^81,82^. It is reported that an increase in the expression of EBP, a key gene functioning in cholesterol biosynthesis, exhibited greater rates of gain and feed efficiency of beef cattle during compensatory growth^83^. Moreover, divergent genes related to cholesterol biosynthesis were upregulated in high efficient chicken^78^. Interestingly, consistent with the above findings the high efficient group had a higher expression level of lincRNA.2988 (fold change = 2.02) and its target gene (fold change = 0.035). B3GALT5 (β-1, 3-Galactosyltransferase) was predicted as a cis target gene of lincRNA.1424. B3GALT, as an O-glucosyltransferase, participate in the elongation of O-fucosylglycan and is involved in fucose metabolic pathway, which is a part of innate immunity glycoproteins (mucins)^84^. Fucose has kept the integrity of the mucosal barrier and has made a relationship between the host tissue and intestinal microbiome in studies related to mice^85^ and rabbits^86^. The fucose metabolism process was positively related to feeding efficiency in bovine, which is reported by Roehe et al. ^87^. Moreover, B3GALT was identified as DEG in Zeng et al. study that conducted a study to identify duodenum genes and pathways through transcriptional profiling in two extreme RFI phenotypes of the duck population^61^. Therefore, overexpression of lincRNA.1424 and B3GALT (fold change = 1.42) in the commercial breed may contribute to the development of the microbiome in the intestine. Also, lincRNA.14534, lincRNA.313, lincRNA.15325, lincRNA.5912, lincRNA.6167, lincRNA.1460 and lincRNA.2104 were up-regulated in the liver of high efficient breed (Ross) and their expression were positively correlated with LRRC8D, LRRC8C^28^, LMAN1^88^, CTDSPL^57^, CDCA7L^89^, CHST7^90^ and AGPAT3^91^. Previous studies reported these target genes to be located within the most significant SNPs associated with RFI or feed efficiency.

Sixteen lincRNAs were found to be co-expressed significantly with the MCHR1 gene. Central nervous system (CNS) through hypothalamic neural circuits integrates peripheral signals related to energy and nutrient status and interprets those to coordinate between feeding behavior and energy expenditure. The previous studies reported that CNS contributes to feed intake regulation with peripheral tissue mechanisms^92^. G protein-coupled receptor (GPCR) family as members of signaling pathways has a crucial role in feeding behavior and energy utilization^93^. Melanin-concentrating hormone receptor (MCHR1) binds MCH with high affinity and the orphan GPCR is a receptor for MCHR1^94^. Therefore, MCHR1 is capable of stimulating various signaling pathways particularly triggering of MAPK/ERK signaling cascade and increase the calcium concentration^95^. Moreover, it has been suggested that MCH might play important roles in the CNS of vertebrates, particularly in the regulation of energy balance in chicken^96^. Therefore, it can be speculated that the overexpression of MCHR1 and its sixteen co-expressed lincRNAs might integrate signals through CNS and peripheral tissue and modulate feeding and energy expenditure, as well affect feed conversion ratio. ADRA2A (encoding adrenoceptor alpha 2A) is a component of the GPCR superfamily and has an important role in the regulation of neurotransmitter release from adrenergic neurons and sympathetic nerves in the central nervous system^97,98^. ADRA2A is involved in different processes of the neurotransmitter, blood pressure, central nervous system and lipolysis^99^. Moreover, ADRA2A is reported to be involved in the fat metabolism pathway. However, the reported SNPs of this gene are associated with increased glucose levels^100^^,^^101^. Mărginean et al. in their study by investigating the ADRA2A gene polymorphisms and mothers–infants' nutritional status, found that defects in the ADRA2A gene might have a dichotomous role, leading to the development of obesity or underweight^101^. Also, a significant lower feed conversion ratio (FCR) was observed in lambs with the CC genotype of the ADRA2A g.1429 C > A mutation rather than those carrying the AA and CA genotypes (0.51 and 0.47, respectively; P < 0.01)^102^. Therefore, ADRA2A by increasing glucose level and fatty acids, probably implicates the lipid metabolism process and energy metabolism. It is well known that lipid and energy metabolism are significant processes in the feed conversion rate of poultry and livestock^103,104^. Zhang et al. by focusing on candidate genes and pathways related to feed efficiency in Hu sheep, reported ADRA2A as candidate gene in feed efficiency^102^. In consistent with these findings, Zhang et al. in their study reported ADRA2A as DEG in the liver of Hu sheep with different residual feed intake^105^. In the present study, ADRA2A was overexpressed in commercial animals and fifteen lincRNA genes were predicted as potential regulators of this gene, in trans mode.

The other known lincRNA (lincRNA.14916) was found to target GAD2, which is associated with stimulating food intake. LincRNA.14916 and its co-expression gene were up-regulated in the commercial breed. The GAD2 gene is responsible for encoding the glutamic acid decarboxylase enzyme (GAD65) and catalyzing the constitution of Gamma-aminobutyric acid (GABA) from L-glutamic acid^106^. Gamma-Aminobutyric acid (GABA) is found in the hypothalamus of birds and mammals and functions as a significant inhibitory neurotransmitter^107^. Several studies have found that stimulating of GABAergic system led to increased food intake in bird and mammals^108,109^. In this regard, GAD2 has found as DEG in a transcriptome analysis of hypothalamus-regulated feed intake^20^. In addition, GAD2 gene variants had significant associations with average daily feed intake (ADFI) in beef cattle^110^. Accordingly, it probably contributes to increase feed conversion ratio and make a balance between feeding and energy expenditure through hypothalamus. As mentioned above, the predicted target genes for the known lincRNAs were similar to the known ilncRNAs, which indicate a synergistic effect between lincRNAs and ilncRNAs for regulating a common biological process. LincRNA.13441 (as a novel lincRNA) was predicted as a potential regulator of 133 genes. Its target genes were enriched in various biological categories including “calcium ion transport” and “muscle contraction regulation”. It is well documented that calcium signaling is an important modulator of lipid metabolism^111^. Hence, the function of these lincRNAs (such as ADRA2A and MCHR1) could be closely related to lipid metabolism as well as feed efficiency development due to their co-expressed targeted mRNAs in the commercial breed affect lipid metabolism.

To better understand how lncRNAs cooperate with their target genes, integrated networks (separately for each of lincRNAs and ilncRNAs) were constructed and one significant module was found in each of the networks. Member genes of both modules were significantly enriched in pathways related to "regulation of lipid catabolic process" (such as ADRA2A) and "translating signals" (such as MCHR1). ADAR2A encodes adrenoceptor alpha 2A and is a regulator of catecholamines, which have been introduced to be associated with energy metabolism and fat metabolism^100,112^. The lipid and energy metabolism were known as significant pathways related to feed conversion rate of poultry and livestock^103,104^. Moreover, this gene was previously found as a significant DEG in sheep with different feed efficiency^102,105^. Furthermore, as discussed earlier, MCHR1 as a receptor for GPCR participates in feeding behavior and energy utilization^94^. These results indicated that the identified lncRNAs might play regulatory roles in the feed efficiency-related terms.

QTL analysis revealed that 11 novel ilncRNAs and 47 novel lincRNAs as putative effective lncRNAs in feed efficiency related processes, as they were overlapped with the potential regions associated with RFI in genome wide. Of these, the predicted target genes of the four lincRNAs including lincRNA.10349 (target ECHDC1)^68^, lincRNA.10336 (target SMAD3)^113^, lincRNA.2986 (target EBP)^83^ and lincRNA.10372 (target TPD52L1)^114^ were related to lipid metabolism. For example, SMAD3 is a multifaceted regulator in adipose physiology, pathogenesis of obesity and type 2 diabetes^113^. As discussed above, animals with lower feed efficiency have higher fat deposition and cholesterol levels^66,67^. In addition, six lincRNAs (linRNA.1964, linRNA.2201, linRNA.2215, linRNA.5649, linRNA.2920, linRNA.2178) were located in more than one QTL related feed efficiency. CTSC, a predicted cis target gene of lincRNA.2201, encodes a lysosomal cysteine proteinase and play a central role in bacterial killing and immune regulation in T lymphocytes. The effects of this gene on the observed difference between the pigs with low and high RFI have been reported^58^. The closest protein coding gene to lincRNA.1964 was DLEU7, which encodes a protein containing 221 amino acids. Fibroblast growth factor (FGF) regulates the expression of DLEU7 during early embryogenesis^56^. Moreover, an association of this gene with human height has been reported^115–117^, which can be suggested to play an important role in chicken growth and feed efficiency. The other gene related to growth rate was RAB30 (cis target of lincRNA.2215). Claire D' Andre et al., conducted a study on the identification and characterization of genes that control fat deposition in chicken^118^. RAB30 was appeared to be down-regulated in slow-growing Xinghua chickens. lincRNAs that were located in QTL regions related to feed efficiency compared with other lincRNAs are more likely to be truly related to feed efficiency. Cis target genes of these lincRNAs that were involved in feed efficiency and RFI or has a known role in the growth rate and lipid metabolism, were interesting functional candidate genes responsible for feed efficiency in chicken. Our findings supported this hypothesis that lncRNAs may affect feed efficiency mainly through regulating lipid metabolism, glucose homeostasis, growth rate, immune system, modification of proteins and energy homeostasis. However further experiments still require to validate the suggested functions of these lncRNAs.

## Conclusion

The present study provided a valuable resource to clarify the genetic basis of feed efficiency and further experiments will corroborate the function of all the RNAs the reported here. To do this end, RNA-Seq data along with bioinformatics approaches were applied and a series of lncRNAs and target genes were identified. Our results indicated that some of the lncRNAs might regulate locally their neighboring genes in cis mode as well as in trans mode. Functional enrichment analysis showed that the identified lncRNAs had enough potential to be related to feed efficiency, as their predicted target genes were significantly involved in the biological processes and KEGG pathways associated with feed efficiency including lipid metabolism, carbohydrate metabolism, and growth. Moreover, several lncRNAs were identified that were overlapped with QTLs controlling feed efficiency such as RFI, which highlighted their importance in this context. Although little is known about the functions of lncRNAs in chicken, our results provided the initial step for studying how changes in lncRNA expression affect the regulation of mechanisms involved in chicken feed efficiency.

## Supplementary Information

Supplementary Information 1.

Supplementary Information 2.

Supplementary Information 3.

Supplementary Information 4.

Supplementary Information 5.

Supplementary Information 6.

Supplementary Information 7.

Supplementary Information 8.

## References

[1] Alsaffar, A. & Khalil, F. Livestock Management LM-589 Why poultry welfare in Kuwait is an obstacle to trade? Sustainable Animal Agriculture for Developing Countries, 711 (2015).

[2] Liu W (2011). A genome-wide SNP scan reveals novel loci for egg production and quality traits in white leghorn and brown-egg dwarf layers. PLoS ONE.

[3] Rekaya R, Sapp RL, Wing T, Aggrey SE (2013). Genetic evaluation for growth, body composition, feed efficiency, and leg soundness. Poult. Sci..

[4] Yousefi Zonuz A, Alijani S, Mohammadi H, Rafat A, Daghigh Kia H (2013). Estimation of genetic parameters for productive and reproductive traits in Esfahan native chickens. J. Livestock Sci. Technol..

[5] Luiting P, Schrama J, Van der Hel W, Urff E (1991). Metabolic differences between White Leghorns selected for high and low residual food consumption. Br. Poult. Sci..

[6] Herd R, Arthur P (2009). Physiological basis for residual feed intake. J. Anim. Sci..

[7] Meale SJ (2017). Exploration of biological markers of feed efficiency in young bulls. J. Agric. Food Chem..

[8] Yang L (2020). Identification of key genes and pathways associated with feed efficiency of native chickens based on transcriptome data via bioinformatics analysis. BMC Genom..

[9] Korostowski L, Sedlak N, Engel N (2012). The Kcnq1ot1 long non-coding RNA affects chromatin conformation and expression of Kcnq1, but does not regulate its imprinting in the developing heart. PLoS Genet.

[10] Liu H, Wang R, Mao B, Zhao B, Wang J (2019). Identification of lncRNAs involved in rice ovule development and female gametophyte abortion by genome-wide screening and functional analysis. BMC Genom..

[11] Hezroni H (2015). Principles of long noncoding RNA evolution derived from direct comparison of transcriptomes in 17 species. Cell Rep..

[12] Meseure D (2016). Prognostic value of a newly identified MALAT1 alternatively spliced transcript in breast cancer. Br. J. Cancer.

[13] Iyer MK (2015). The landscape of long noncoding RNAs in the human transcriptome. Nat. Genet..

[14] Sui Y, Han Y, Zhao X, Li D, Li G (2019). Long non-coding RNA Irm enhances myogenic differentiation by interacting with MEF2D. Cell Death Dis..

[15] Li Z (2017). Integrated analysis of long non-coding RNAs (LncRNAs) and mRNA expression profiles reveals the potential role of LncRNAs in skeletal muscle development of the chicken. Front. Physiol..

[16] Muret K (2017). Long noncoding RNA repertoire in chicken liver and adipose tissue. Genet. Sel. Evol..

[17] Liu Y (2017). Analyses of Long Non-Coding RNA and mRNA profiling using RNA sequencing in chicken testis with extreme sperm motility. Sci. Rep..

[18] Kern C (2018). Genome-wide identification of tissue-specific long non-coding RNA in three farm animal species. BMC Genom..

[19] Yi, G. et al. In-depth duodenal transcriptome survey in chickens with divergent feed efficiency using RNA-Seq. PloS one 10, e0136765 (2015). 10.1371/journal.pone.0136765 PMC4721924 26418546

[20] Li H (2018). Transcriptome profile analysis reveals an estrogen induced LncRNA associated with lipid metabolism and carcass traits in chickens (Gallus Gallus). Cell. Physiol. Biochem..

[21] Ren T (2018). Sequencing and characterization of lncRNAs in the breast muscle of Gushi and Arbor Acres chickens. Genome.

[22] Tang R (2020). Comprehensive analysis of lncRNA and mRNA expression changes in Tibetan chicken lung tissue between three developmental stages. Anim. Genet..

[23] Li W (2020). Analysis of four complete linkage sequence variants within a novel lncRNA located in a growth QTL on chromosome 1 related to growth traits in chickens. J. Anim. Sci..

[24] Ning C (2020). Long non-coding RNA and mRNA profile of liver tissue during four developmental stages in the chicken. Front. Genet..

[25] Cao C (2017). Impact of exudative diathesis induced by selenium deficiency on LncRNAs and their roles in the oxidative reduction process in broiler chick veins. Oncotarget.

[26] Yang J, Gong Y, Cai J, Liu Q, Zhang Z (2019). lnc-3215 suppression leads to calcium overload in selenium deficiency-induced chicken heart lesion via the lnc-3215-miR-1594-TNN2 pathway. Mol. Therapy – Nucl. Acids.

[27] Fonseca LD (2019). Liver proteomics unravel the metabolic pathways related to feed efficiency in beef cattle. Sci. Rep..

[28] Brunes LC (2021). Weighted single-step genome-wide association study and pathway analyses for feed efficiency traits in Nellore cattle. J. Anim. Breed. Genet..

[29] Izadnia HR, Tahmoorespur M, Bakhtiarizadeh MR, Nassiri M, Esmaeilkhanien S (2019). Gene expression profile analysis of residual feed intake for Isfahan native chickens using RNA-SEQ data. Ital. J. Anim. Sci..

[30] Andrews, S. A quality control tool for high throughput sequence data, 2010).

[31] Bolger AM, Lohse M, Usadel B (2014). Trimmomatic: a flexible trimmer for Illumina sequence data. Bioinformatics.

[32] Dobin A (2013). STAR: ultrafast universal RNA-seq aligner. Bioinformatics.

[33] Pertea M (2015). StringTie enables improved reconstruction of a transcriptome from RNA-seq reads. Nat. Biotechnol..

[34] Trapnell C (2010). Transcript assembly and quantification by RNA-Seq reveals unannotated transcripts and isoform switching during cell differentiation. Nat. Biotechnol..

[35] Vance KW, Ponting CP (2014). Transcriptional regulatory functions of nuclear long noncoding RNAs. Trends Genet..

[36] Wang Y (2018). Genome-wide identification and characterization of putative lncRNAs in the diamondback moth, Plutella xylostella (L.). Genomics.

[37] Kuo RI (2017). Normalized long read RNA sequencing in chicken reveals transcriptome complexity similar to human. BMC Genom..

[38] Bakhtiarizadeh MR, Salami SA (2019). Identification and expression analysis of long noncoding RNAs in fat-tail of sheep breeds. G3 Genes Genomes Genetics.

[39] Bateman A (2004). The Pfam protein families database. Nucl. Acids Res..

[40] Kang Y-J (2017). CPC2: a fast and accurate coding potential calculator based on sequence intrinsic features. Nucl. Acids Res..

[41] Sun L (2013). Utilizing sequence intrinsic composition to classify protein-coding and long non-coding transcripts. Nucl. Acids Res..

[42] Wang L (2013). CPAT: Coding-potential assessment tool using an alignment-free logistic regression model. Nucl. Acids Res..

[43] Li A, Zhang J, Zhou Z (2014). PLEK: a tool for predicting long non-coding RNAs and messenger RNAs based on an improved k-mer scheme. BMC Bioinform..

[44] Wucher V (2017). FEELnc: A tool for long non-coding RNA annotation and its application to the dog transcriptome. Nucl. Acids Res..

[45] Foissac, S. et al. Livestock genome annotation: transcriptome and chromatin structure profiling in cattle, goat, chicken and pig. bioRxiv, 316091, 10.1101/316091 (2018).

[46] Ulitsky I (2016). Evolution to the rescue: using comparative genomics to understand long non-coding RNAs. Nat. Rev. Genet..

[47] Muret K (2019). Long noncoding RNAs in lipid metabolism: literature review and conservation analysis across species. BMC Genomics.

[48] Kanehisa M, Furumichi M, Sato Y, Ishiguro-Watanabe M, Tanabe M (2021). KEGG: Integrating viruses and cellular organisms. Nucleic Acids Res..

[49] Kuleshov MV (2016). Enrichr: A comprehensive gene set enrichment analysis web server 2016 update. Nucl. Acids Res..

[50] Hu Z-L, Park CA, Reecy JM (2016). Developmental progress and current status of the Animal QTLdb. Nucl. Acids Res..

[51] Mering CV (2003). STRING: A database of predicted functional associations between proteins. Nucl. Acids Res..

[52] Shannon P (2003). Cytoscape: A software environment for integrated models of biomolecular interaction networks. Genome Res..

[53] Nepusz T, Yu H, Paccanaro A (2012). Detecting overlapping protein complexes in protein-protein interaction networks. Nat. Methods.

[54] Untergasser A (2007). Primer3Plus, an enhanced web interface to Primer3. Nucl. Acids Res..

[55] Kuang J, Yan X, Genders AJ, Granata C, Bishop DJ (2018). An overview of technical considerations when using quantitative real-time PCR analysis of gene expression in human exercise research. PLoS ONE.

[56] Zhu X (2012). Characterization and expressional analysis of Dleu7 during Xenopus tropicalis embryogenesis. Gene.

[57] Wolc A (2013). Pedigree and genomic analyses of feed consumption and residual feed intake in laying hens. Poult. Sci..

[58] Gondret F (2017). A transcriptome multi-tissue analysis identifies biological pathways and genes associated with variations in feed efficiency of growing pigs. BMC Genom..

[59] Alexandre PA (2020). Exploring the regulatory potential of long non-coding RNA in feed efficiency of indicine cattle. Genes.

[60] Kern C (2018). Genome-wide identification of tissue-specific long non-coding RNA in three farm animal species. BMC Genom..

[61] Zhang T (2017). Analysis of long noncoding RNA and mRNA using RNA sequencing during the differentiation of intramuscular preadipocytes in chicken. PLoS ONE.

[62] Zhu B, Xu M, Shi H, Gao X, Liang P (2017). Genome-wide identification of lncRNAs associated with chlorantraniliprole resistance in diamondback moth Plutella xylostella (L.). BMC Genom..

[63] Wang L (2018). Genome-wide identification and characterization of novel lncRNAs in Ginkgo biloba. Trees.

[64] Bakhtiarizadeh MR, Hosseinpour B, Arefnezhad B, Shamabadi N, Salami SA (2016). In silico prediction of long intergenic non-coding RNAs in sheep. Genome.

[65] Jandura A, Krause HM (2017). The New RNA World: Growing evidence for long noncoding RNA functionality. Trends Genet..

[66] Karisa B, Moore S, Plastow G (2014). Analysis of biological networks and biological pathways associated with residual feed intake in beef cattle. Anim. Sci. J..

[67] Nafikov RA, Beitz DC (2007). Carbohydrate and Lipid Metabolism in Farm Animals. J. Nutr..

[68] Linster CL (2011). Ethylmalonyl-CoA Decarboxylase, a New Enzyme Involved in Metabolite Proofreading*. J. Biol. Chem..

[69] Schweiger M (2014). Measurement of lipolysis. Methods Enzymol..

[70] Hue L, Taegtmeyer H (2009). The Randle cycle revisited: a new head for an old hat. Am. J. Physiol.-Endocrinol. Metabolism.

[71] Resnyk CW (2017). Transcriptional analysis of abdominal fat in chickens divergently selected on bodyweight at two ages reveals novel mechanisms controlling adiposity: validating visceral adipose tissue as a dynamic endocrine and metabolic organ. BMC Genom..

[72] Yang R-Y (2016). Identification of VPS13C as a galectin-12-binding protein that regulates galectin-12 protein stability and adipogenesis. PLoS ONE.

[73] Lemley C, Butler S, Butler W, Wilson M (2008). Insulin alters hepatic progesterone catabolic enzymes cytochrome P450 2C and 3A in dairy cows. J. Dairy Sci..

[74] Harmon JS, Sheridan MA (1992). Glucose-stimulated lipolysis in rainbow trout, Oncorhynchus mykiss, liver. Fish Physiol. Biochem..

[75] Zhuo Z, Lamont SJ, Lee WR, Abasht B (2015). RNA-seq analysis of abdominal fat reveals differences between modern commercial broiler chickens with high and low feed efficiencies. PLoS ONE.

[76] Bu G (2013). Characterization of the novel duplicated PRLR gene at the late-feathering K locus in Lohmann chickens. J. Mol. Endocrinol..

[77] Hou X (2020). Transcriptome analysis of skeletal muscle in pigs with divergent residual feed intake phenotypes. DNA Cell Biol..

[78] Abasht B, Zhou N, Lee WR, Zhuo Z, Peripolli E (2019). The metabolic characteristics of susceptibility to wooden breast disease in chickens with high feed efficiency. Poult. Sci..

[79] Mukiibi R (2018). Transcriptome analyses reveal reduced hepatic lipid synthesis and accumulation in more feed efficient beef cattle. Sci. Rep..

[80] Yang F, Wang Q, Wang M, He K, Pan Y (2012). Associations between gene polymorphisms in two crucial metabolic pathways and growth traits in pigs. Chin. Sci. Bull..

[81] Incardona JP, Eaton S (2000). Cholesterol in signal transduction. Curr. Opin. Cell Biol..

[82] Ohvo-Rekilä H, Ramstedt B, Leppimäki P, Slotte JP (2002). Cholesterol interactions with phospholipids in membranes. Prog. Lipid Res..

[83] Connor EE (2010). Enhanced mitochondrial complex gene function and reduced liver size may mediate improved feed efficiency of beef cattle during compensatory growth. Funct. Integr. Genom..

[84] Hooper LV, Xu J, Falk PG, Midtvedt T, Gordon JI (1999). A molecular sensor that allows a gut commensal to control its nutrient foundation in a competitive ecosystem. Proc. Natl. Acad. Sci..

[85] Hoorens PR (2011). Genome wide analysis of the bovine mucin genes and their gastrointestinal transcription profile. BMC Genomics.

[86] Pacheco AR (2012). Fucose sensing regulates bacterial intestinal colonization. Nature.

[87] Roehe, R. et al. Bovine host genetic variation influences rumen microbial methane production with best selection criterion for low methane emitting and efficiently feed converting hosts based on metagenomic gene abundance. PLoS genetics 12, e1005846 (2016). 10.1371/journal.pgen.1005846 PMC4758630 26891056

[88] Reyer H (2017). Strategies towards improved feed efficiency in pigs comprise molecular shifts in hepatic lipid and carbohydrate metabolism. Int. J. Mol. Sci..

[89] Ramayo-Caldas Y (2019). Integrating genome-wide co-association and gene expression to identify putative regulators and predictors of feed efficiency in pigs. Genet. Sel. Evol..

[90] Dawson PA, Gardiner B, Grimmond S, Markovich D (2006). Transcriptional profile reveals altered hepatic lipid and cholesterol metabolism in hyposulfatemic NaS1 null mice. Physiol. Genomics.

[91] Zarek CM, Lindholm-Perry AK, Kuehn LA, Freetly HC (2017). Differential expression of genes related to gain and intake in the liver of beef cattle. BMC. Res. Notes.

[92] Richards M, Proszkowiec-Weglarz M (2007). Mechanisms regulating feed intake, energy expenditure, and body weight in poultry. Poult. Sci..

[93] Schioth, H. B. G protein-coupled receptors in regulation of body weight. CNS & Neurological Disorders-Drug Targets (Formerly Current Drug Targets-CNS & Neurological Disorders) 5, 241–249 (2006). 10.2174/187152706777452263 16787226

[94] Bächner D, Kreienkamp H-J, Weise C, Buck F, Richter D (1999). Identification of melanin concentrating hormone (MCH) as the natural ligand for the orphan somatostatin-like receptor 1 (SLC-1). FEBS Lett..

[95] Hawes BE (2000). The melanin-concentrating hormone receptor couples to multiple G proteins to activate diverse intracellular signaling pathways. Endocrinology.

[96] Tritos N, Maratos-Flier E (1999). Two important systems in energy homeostasis: melanocortins and melanin-concentrating hormone. Neuropeptides.

[97] Manca A (1997). Detailed physical analysis of a 1.5-megabase YAC contig containing the MXI1 and ADRA2A genes. Genomics.

[98] Fagerholm V (2004). Altered glucose homeostasis in α2A-adrenoceptor knockout mice. Eur. J. Pharmacol..

[99] Kaabi B (2016). ADRA2A Germline Gene Polymorphism is Associated to the Severity, but not to the Risk, of Breast Cancer. Pathology & Oncology Research.

[100] Sohani, Z. N. et al. Risk alleles in/near ADCY5, ADRA2A, CDKAL1, CDKN2A/B, GRB10, and TCF7L2 elevate plasma glucose levels at birth and in early childhood: results from the FAMILY study. PloS One 11, e0152107 (2016). 10.1371/journal.pone.0152107 PMC4822946 27049325

[101] Mărginean CO (2019). The relationship between MMP9 and ADRA2A gene polymorphisms and mothers–newborns’ nutritional status: an exploratory path model (STROBE compliant article). Pediatr. Res..

[102] Zhang D (2019). Transcriptome analysis identifies candidate genes and pathways associated with feed efficiency in hu sheep. Front. Genet..

[103] Xu C (2020). A transcriptome analysis reveals that hepatic glycolysis and lipid synthesis are negatively associated with feed efficiency in DLY pigs. Sci. Rep..

[104] Kaewpila C, Sommart K, Mitsumori M (2018). Dietary fat sources affect feed intake, digestibility, rumen microbial populations, energy partition and methane emissions in different beef cattle genotypes. Animal.

[105] Zhang, D. et al. Transcriptome analysis of long noncoding RNAs ribonucleic acids from the livers of Hu sheep with different residual feed intake. Animal 15, 100098 (2021). 10.1016/j.animal.2020.100098 33573993

[106] Erdö SL, Wolff JR (1990). γ-Aminobutyric acid outside the mammalian brain. J. Neurochem..

[107] Dicken MS, Hughes AR, Hentges ST (2015). Gad1 mRNA as a reliable indicator of altered GABA release from orexigenic neurons in the hypothalamus. Eur. J. Neurosci..

[108] Tajalli S, Jonaidi H, Abbasnejad M, Denbow D (2006). Interaction between nociceptin/orphanin FQ (N/OFQ) and GABA in response to feeding. Physiol. Behav..

[109] Bungo T (2003). Intracerebroventricular injection of muscimol, baclofen or nipecotic acid stimulates food intake in layer-type, but not meat-type, chicks. Brain Res..

[110] Guo H (2008). Mapping and association of GAD2 and GIP gene variants with feed intake and carcass traits in beef cattle. Arch. Anim. Breed..

[111] Xue B, Greenberg AG, Kraemer FB, Zemel MB (2001). Mechanism of intracellular calcium ([Ca2+]i) inhibition of lipolysis in human adipocytes. FASEB J..

[112] Lima JJ (2007). Association analyses of adrenergic receptor polymorphisms with obesity and metabolic alterations. Metabolism.

[113] Yadav H (2011). Protection from Obesity and Diabetes by Blockade of TGF-β/Smad3 Signaling. Cell Metab..

[114] Kamili A (2015). TPD52 expression increases neutral lipid storage within cultured cells. J. Cell Sci..

[115] Kang SJ (2010). Genome-wide association of anthropometric traits in African- and African-derived populations. Hum. Mol. Genet..

[116] Sovio U (2009). Genetic Determinants of Height Growth Assessed Longitudinally from Infancy to Adulthood in the Northern Finland Birth Cohort 1966. PLoS Genet..

[117] Weedon MN (2008). Genome-wide association analysis identifies 20 loci that influence adult height. Nat. Genet..

[118] Claire D’Andre H (2013). Identification and characterization of genes that control fat deposition in chickens. Journal of Animal Science and Biotechnology.

